# Genome-Wide Identification and Characterization of *RopGEF* Gene Family in C_4_ Crops

**DOI:** 10.3390/genes15091112

**Published:** 2024-08-23

**Authors:** Xiuqing Jing, Ning Deng, Yongduo Cai

**Affiliations:** 1 College of Biological Sciences and Technology, Taiyuan Normal University, Jinzhong 030619, China; ddd_denny@163.com (N.D.); 15513996162@163.com (Y.C.); 2 Shanxi Key Laboratory of Earth Surface Processes and Resource Ecology Security in Fenhe River Basin, Taiyuan Normal University, Jinzhong 030619, China; 3 College of Life Science, Shanxi University, Taiyuan 030006, China

**Keywords:** RopGEF, C_4_ Crops, phylogenetic analysis, gene expression

## Abstract

In plants, RopGEF-mediated ROP signaling is pivotal in cellular signaling pathways, including apical growth, pollen germination and perception, intercellular recognition, as well as in responses to biotic and abiotic stresses. In this study, we retrieved a total of 37 RopGEF members from three C_4_ Crops, of which 11 are from millet, 11 from sorghum, and 15 from maize. Based on their phylogenetic relationships and structural characteristics, all RopGEF members are classified into four subfamilies. The qRT-PCR technique was utilized to evaluate the expression profiles of 11 *SiRopGEFs* across different tissues in foxtail millet. The findings indicated that the majority of the *SiRopGEFs* exhibited higher expression levels in leaves as opposed to roots and stems. The levels of expression of *SiRopGEF* genes were examined in response to abiotic stress and plant hormones. *SiRopGEF1*, *SiRopGEF5*, *SiRopGEF6*, and *SiRopGEF8* showed significant induction under abiotic stresses such as salt, cold, and heat. On the other hand, *SiRopGEF1*, *SiRopGEF2*, and *SiRopGEF7* were consistently upregulated, while *SiRopGEF3*, *SiRopGEF4*, *SiRopGEF6*, *SiRopGEF9*, and *SiRopGEF10* were downregulated upon exposure to abscisic acid (ABA), ethylene (ET), salicylic acid (SA), and gibberellic acid (GA_3_) hormones. The alterations in the expression patterns of RopGEF members imply their potential functions in plant growth and development, abiotic stress response, and hormone signal transduction. These discoveries suggest that the *RopGEF* genes may function as a potential genetic marker to facilitate future studies in elucidating the functional characteristics of RopGEFs.

## 1. Introduction

GTP-binding proteins (G proteins) are crucial molecular switches in eukaryotic signal transduction [1,2]. Two major classes of signaling G proteins are recognized: the Ras superfamily of monomeric small GTPases, and heterotrimeric G proteins [3]. The Ras superfamily consists of five smaller, evolutionarily conserved subfamilies (Ras, Rho, Rab, Arf, and Ran) [3]. In plants, the small GTP-binding proteins named ROPs [4], a plant-specific subfamily of RHO GTPases, also known as RACs, function as signaling switches that control diverse biological processes including cell polarity establishment, tip growth, morphogenesis, hormone responses, and numerous other cellular processes [5,6,7].

As signaling switches, ROPs activate signaling pathways by switching from a GDP-bound inactive to a GTP-bound active conformation [1,2]. The activity of ROPs is positively regulated by guanine nucleotide exchange factors (GEFs), which stimulate ROPs by catalyzing the exchange of GDP with GTP [8]. In plants, three classes of GEFs with characteristic catalytic GEF domains can activate ROPs: SPIKE contains a DOCK domain [9], switch-associated protein 70 (SWAP70) contains a DBL domain [10], and RopGEF contains a plant-specific ROP nucleotide exchanger PRONE domain [7]. Additionally, the activity of ROPs is negatively regulated by GTPase-activating proteins (GAPs) and guanine nucleotide dissociation inhibitors (GDIs) [1]. GAPs deactivate ROPs by facilitating the hydrolysis of ROPs from GTP to GDP, and GDIs can sequester ROP in the cytosol to ensure its inactivation [1].

Currently, RopGEFs have been identified in multiple species, and the functions of some of their members have been investigated. In *Arabidopsis thaliana,* a total of 14 members of the RopGEF family have been identified [8,11], and some of them have been studied as being involved in plant growth, fertilization, immune response and other aspects [12,13,14,15,16,17,18]. AtRopGEF1 and AtRopGEF10 are involved in the polar growth of cells, the elongation of pollen tubes [12], the transport of polar auxin, the root gravitropic response [13], the formation and movement of stomata, and the process of response to the plant hormone ABA [15]. AtRopGEF2 act as a negative regulator in phytochrome B (phyB)-mediated red light-induced stomatal opening, and can interact with AtROP7 and AtROP2 to enhance their intrinsic nucleotide exchange rates [14]. AtRopGEF4 functioned redundantly with AtRopGEF2 in red light-induced stomatal opening [14]. AtRopGEF4 is also involved in regulating stomatal development and the ABA signaling pathway [15]. AtRopGEF7 participates in the regulation of root tip stem cell homeostasis [16]. AtRopGEF8/12 plays a crucial role in the polarized growth of pollen tubes by interacting with the pollen receptor kinase AtPRK6 [18]. AtRopGEF11 plays a key role in the regulation of light signals and the maintenance of normal root development [17].

In rice (*Oryza sativa*), a total of 11 members of the RopGEF family have been identified [19], and it has been demonstrated that OsRopGEFs are involved in pollen germination, pollen tube growth, plant growth, and development [19,20,21,22]. OsRopGEF1 participates in drought stress tolerance by interacting with OsRbohA and OsCBM1 to regulate ROS production [22]. OsRopGEF3 plays a key role in regulating root hair elongation and ROS production by interacting with OsRac3, which interacts with OsRBOH5 to generate ROS in rice [20]. OsRopGEF7B functions in the development of flower organs, thus influencing the seed setting rate of rice [23]. OsRopGEF10 can activate the development of small cuticular papillae (SP) on leaf surfaces by activating OsRac1 to turn on the molecular signaling pathway for SP development [24]. Moreover, the double-knockout mutants *OsRopGEF2* and *OsRopGEF8* were characterized by significantly reduced pollen germination and seed yield, suggesting that *OsRopGEF2* and *OsRopGEF8* may play a key role in pollen germination and pollen tube growth [19].

There were 10 members of the RopGEF family identified in *Medicago truncatula* [25]. MtRopGEF2 is one member of the MtRopGEF family and is involved in the regulation of root hair development and polar growth through interaction with MtRops [25]. The activity of MtRopGEF7 depends on the N^68^ and R^76^ residues of ROPs [26]. In *Brassica rapa*, a total of 21 BrRopGEFs were identified, and *BrRopGEF9* and *BrRopGEF13* may play significant roles in regulating abiotic stress tolerance [27]. In *Physcomitrium patens*, RopGEFs and GAPs form membrane domains when they grow at the tips of cells [28].

In certain plants, the initial product of carbon dioxide fixation is oxaloacetate, a four-carbon compound. Plants utilizing this carbon fixation pathway, such as millet, sorghum, and maize, are referred to as C_4_ plants. The mechanism of C_4_ photosynthesis gives plants a competitive advantage under conditions of high light intensity, high temperature, and limited water supply, meaning they can better regulate water balance than most C_3_ plants. To date, there have been no investigations concerning the members of the RopGEFs family in C_4_ plants, and the biological roles of these members within C_4_ plants remain unexplored.

Foxtail millet (*Setaria italica*) is one of the main cereal crops, possessing excellent nutritional properties. The seeds of millet are rich in protein and have a high content of essential amino acids. The germplasm resources of millet are abundant, featuring the largest number of cultivated and wild types, which holds a favorable application prospect in crop improvement projects such as gene mapping, allelic gene mining, and excellent variety breeding. Sorghum (*Sorghum bicolor*) is also a type of cereal crop, known for its strong drought resistance and salt tolerance. It is mainly grown in arid and saline-alkali areas. Sorghum has a wide range of uses, especially in brewing, animal feed, and industrial starch, and in recent years, sorghum has also attracted attention as a high-quality energy plant. Maize (*Zea mays*), is one of the most widely distributed food crops in the world, playing an important role in industries such as grain, feed, and processing. Thus, the RopGEF family members of foxtail millet, sorghum, and maize were successfully identified and characterized through bioinformatic techniques in this study. Furthermore, the expression patterns of *SiRopGEFs* were systematically examined using qRT-PCR. These outcomes will support future research on the biological functions of *RopGEF* gene family members in C_4_ Crops.

## 2. Materials and Methods

### 2.1. Identification of RopGEF Family Members in Foxtail Millet

The RopGEF family members of *Arabidopsis* have been previously documented [8]. We utilized the RopGEF protein sequences obtained from the *Arabidopsis* Information Resource (TAIR) database (http://www.arabidopsis.org) (accessed on 30 June 2023) as queries for BLASTP search in order to acquire RopGEF sequences of millet, sorghum, and maize from the Ensembl database (http://plants.ensembl.org/index.html) (accessed on 3 July 2023). Initially, the candidate proteins of millet, sorghum and maize were preliminary authenticated by a BLASTP search. Subsequently, the SMART (http://smart.embl-heidelberg.de/) (accessed on 27 October 2023), InterPro (http://www.ebi.ac.uk/interpro/) (accessed on 27 October 2023), and Conserved Domain Database (CDD) (http://www.ncbi.nlm.nih.gov/cdd/) (accessed on 29 October 2023) were employed for scanning to identify the presence of PRONE (PFAM03759) domains in the candidate proteins. The physicochemical properties of RopGEF family proteins, including molecular weight (Da), isoelectric point (pI), aliphatic index (AI), instability index (II), major amino acids, and grand average of hydropathy (GRAVY), were analyzed using the ProtParam (https://www.expasy.org/resources/protparam) (accessed on 12 November 2023).

### 2.2. Distribution of Genes on Chromosomes

The location of the 11 *SiRopGEF* genes, 11 *SbRopGEF* genes, and 15 *ZmRopGEF* genes were mapped on the nine chromosomes of millet genomes, the ten chromosomes of sorghum genomes, and the ten chromosomes of maize genomes, correspondingly, based on the annotation information available on the Ensembl Plants website (https://plants.ensembl.org/Setaria_italica/Info/Index) (accessed on 5 July 2023). The map was drawn by the Mapchart software 2.32 (http://www.wageningenur.nl/en.htm) (accessed on 5 July 2023).

### 2.3. Organization of Exons and Introns, Conserved Amino Acid Motif Arrangement, and Three-Dimensional (3D) Folding Structure Prediction

The exon/intron structures of RopGEF family genes were determined based on alignments of their coding sequences with corresponding genomic sequences. The gene structure diagram, which shows both exon position and gene length, was created using the Gene Structure Display Server (GSDS: https://gsds.gao-lab.org/) (accessed on 17 November 2023). Conserved amino acid motif arrangement was determined using the Multiple Expectation for Motif Elicitation (MEME) tool (http://meme-suite.org/) (version 4.9.1) (accessed on 13 November 2023) online. The Phyre2 website (http://www.sbg.bio.ic.ac.uk/phyre2) (accessed on 19 November 2023) was utilized to predict the three-dimensional structure of RopGEF proteins to investigate structural modifications and their effects on the functions of RopGEF family members.

### 2.4. Phylogenetic Relationship

The candidate RopGEF proteins were initially multiply aligned by using the ClustalW v2.0 online tool (http://www.ebi.ac.uk/Tools/webservices/services/msa/clustalw2_soap) (accessed on 26 July 2023) to investigate the evolutionary relationships within the RopGEF family. Subsequently, the maximum likelihood phylogenetic tree was constructed using MEGA 11.0.10 software with default parameters, and the reliability of interior branches was evaluated with 1000 bootstrap repetitions.

### 2.5. Plant Material, Growth Conditions, Abiotic Stresses and Hormonal Applications in Foxtail Millet

In 2023, the millet (Yugu 1) was cultivated in the plant culture room located at the farm at Taiyuan Normal University. The millet plants were nurtured in a seedling tray filled with a mixture of soil and vermiculite (1:1), with a cycle of 16 h at 25 °C during the day and 8 h at 20 °C at night, while maintaining relative humidity at approximately 75%. The millet plants at the seedling stage (28 days) were exposed to various abiotic stresses and phytohormones. For salt stress, millet seedlings were irrigated with a 200 mM NaCl solution, and leaves were collected at intervals of 0, 0.5, 1, 3, 6, 12, and 24 h following the irrigation. The entire millet plants were exposed to a high temperature condition of 40 °C or a cryogenic temperature condition of 4 °C, and leaves were collected at intervals of 0, 0.5, 1, 3, 6, 12, and 24 h. For hormonal treatments, plant hormones such as 100 μM ABA, 500 μM SA, 100 μM GA_3_, and 100 μM ET, were evenly sprayed on the leaf surfaces of millet seedlings, and the leaves were harvested at intervals of 0, 0.5, 1, 3, 6, 12, and 24 h following the application.

The different plant organs (root, stem, and leaf) of the millet plants at the seedling stage (28 days) were also collected for analyzing the tissue-specific expression of SiRopGEF family genes. The samples were promptly frozen in liquid nitrogen and stored at −80 °C for subsequent analysis.

### 2.6. Total RNA Extraction, cDNA Reverse Transcription, and qRT-PCR Analysis

Total RNA was extracted from millet leaves by utilizing TRIzol reagents (Invitrogen, Waltham, MA, USA). Subsequently, cDNA reverse transcription was performed on 2 μg of total RNA in 25 µL reaction systems using the M-MLV First Strand Kit (Invitrogen). The primers for quantitative real-time PCR (qRT-PCR) were designed based on *SiRopGEF* sequences using Primer 6.0 (Table S3). The qRT-PCR analysis was conducted in an Applied Biosystems Quantitative Real-Time PCR Detection System. The millet actin gene (*SiActin1*, Transcript ID: Si026509m) was employed as an internal control to standardize all mRNA transcriptional levels.

### 2.7. Statistical Analysis

An analysis of variance was conducted on the data. The mean and standard deviation of the three replicates for each treatment were compared, utilizing the SPSS 11.5 software package (SPSS, Chicago, IL, USA), and employing the least significance difference (LSD) test at a significance level of 5%. Graphs were created using Origin 8.5.

## 3. Results

### 3.1. Identification and Annotation of RopGEF Family Members

The *Arabidopsis RopGEF* genes were utilized as queries sequences against the Hidden Markov Model (HMM) algorithm [27] to retrieve and characterize the *RopGEF* gene family members in three studied C_4_ Crops (millet, sorghum and maize). A total of 37 *RopGEF* genes were identified in the three studied C_4_ Crops. The number of *RopGEF* gene members varies among these plants, such as 11, 11, and 15 *RopGEF* genes from millet, sorghum, and maize, respectively (Table 1). The potential domains of the RopGEF family members were confirmed through the conserved domain database, Pfam, and SMART databases. The results indicated that all the RopGEF proteins possess a conserved PRONE (PFAM03759) domain for GEF catalytic activity but have variable *N*-terminal and *C*-terminal domains (Figure S1).

**Figure 1:**
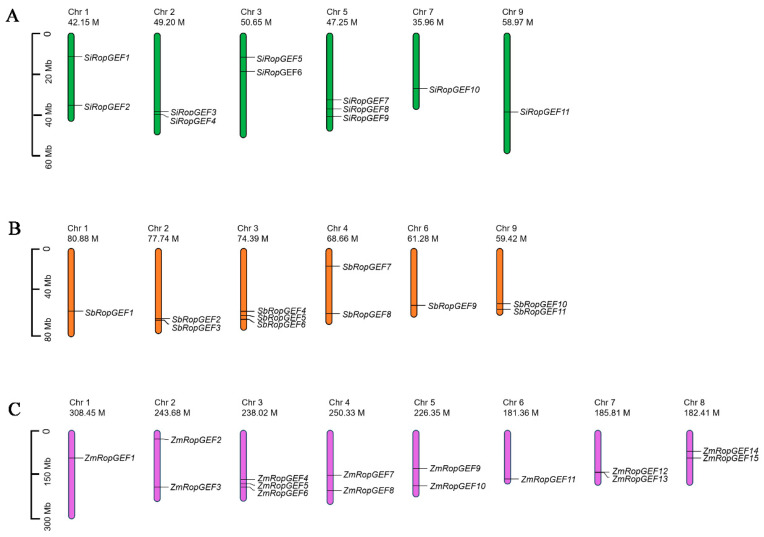
Chromosome distribution of the 37 *RopGEF* genes. Chromosome location map drawn with the Mapchart software. The scale is in megabases (Mb). (**A**) The distribution of 11 *SiRopGEF* genes on chromosomes. (**B**) The distribution of 11 *SbRopGEF* genes on chromosomes. (**C**) The distribution of 15 *ZmRopGEF* genes on chromosomes.

The RopGEF family genes were mapped onto the chromosomes based on their location information on chromosomes in the genomes (Table 1 and Figure 1). We noted that all 11 *SiRopGEFs* are distributed on six of the nine chromosomes in the millet genome (Figure 1A). The majority of *SiRopGEFs* (three genes) have been mapped on chromosome 5, while two *SiRopGEFs* were found on chromosomes 1, 2, and 3, and one *SiRopGEF* was found on chromosome 7 and 9. All 11 *SbRopGEFs* are mapped on eight of the ten chromosomes in the sorghum genome (Figure 1B). Three *SiRopGEFs* were found on chromosome 3, while each chromosome 2, 4, and 9 comprises two genes, and only one gene was found on chromosome 1 and 6. In the maize genome, 15 *ZmRopGEFs* are distributed on six of the ten chromosomes (Figure 1C). There are 3 *ZmRopGEFs* on chromosome 3, and 2 *ZmRopGEFs* on chromosomes 2, 4, 5, 7, and 9. And there is only one *ZmRopGEF* gene on chromosomes 1 and 6. These identified *RopGEFs* are named according to their chromosome locations in their genomes (Table 1 and Figure 1). 

Moreover, the physiochemical characteristics and amino acid sequence of RopGEF family members were investigated through the EXPASY PROTPARAM (https://web.expasy.org/protparam/) online tool. The putative length of the RopGEF proteins and molecular weights vary widely, ranging from 47,376.1 Da (ZmRopGEF7) to 63,440.64 Da (SbRopGEF10) in the three studied C_4_ Crops. The majority of C_4_ RopGEF proteins studied were acidic in nature based on their isoelectric point, which is lower than seven (Table S1). However, the isoelectric point of some RopGEF members (SbRopGEF11, ZmRopGEF7, and ZmRopGEF14) was greater than seven, indicating that they are alkaline proteins in nature (Table S1). These identified RopGEF proteins are stable proteins because the instability index of all members is <40 (Table S1). All the RopGEF proteins are found to be hydrophilic based on their GRAVY value (Table S1). Furthermore, the aliphatic index values range from the lowest 73.74 (SiRopGEF4) to the highest 92.86 (ZmRopGEF7). The predominant amino acid of the RopGEF proteins is Ser, followed by Leu, while the other most abundant amino acids are Ala, Glu, Asp, Arg, Glu, or Lys, which vary depending on the individual RopGEF (Table S1).

### 3.2. Phylogenetic Analysis and Classification of the RopGEF Family Members

**Figure 2:**
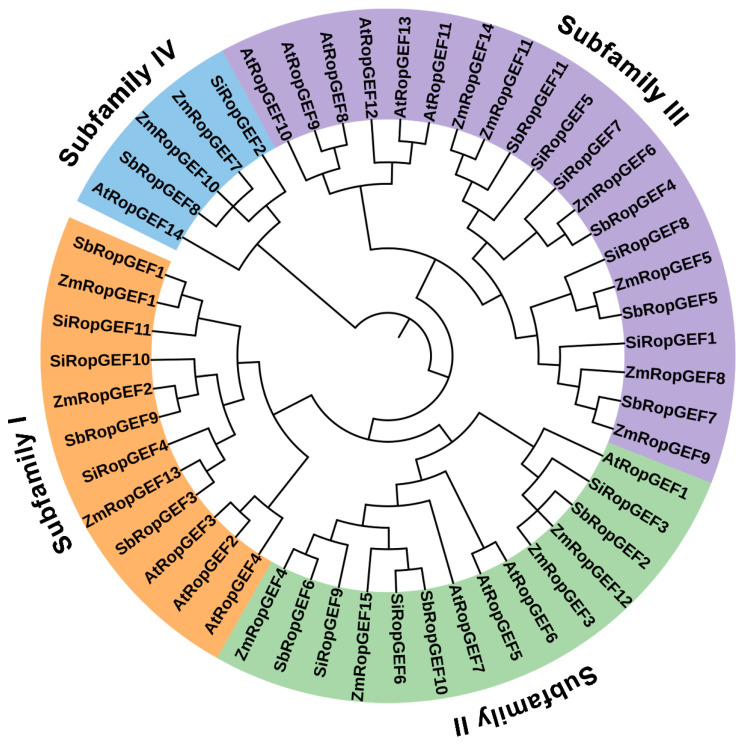
Phylogenetic analysis of RopGEF family members from millet, sorghum, maize, and *Arabidopsis*. The phylogenetic tree was created using complete protein sequences and the neighbor-joining (NJ) method. Various color shades were employed to differentiate between different Clades, while Subfamilies I–IV were used to classify the RopGEF family members.

In order to investigate the phylogenetic relationships of these RopGEF family members and predict the possible biological functions of RopGEF proteins, we analyzed the phylogenetic relationships of 37 identified RopGEFs from three C_4_ plants (millet, sorghum and maize) and 14 AtRopGEFs from *Arabidopsis* (Figure 2). The results demonstrated that the 51 RopGEF proteins in the phylogenetic tree were divided into four subfamilies (Figure 2). For SiRopGEFs, there are three members each in group I (SiRopGEF4, -10, -11) and group II (SiRopGEF3, -6, -9), four members in subfamily III (SiRopGEF1, -5, -7, -8), and one member in group IV (SiRopGEF2). SbRopGEFs and SiRopGEFs exhibit a similar distribution of members in phylogenetic tree. Specifically, three members are in group I (SbRopGEF1, -3, -9) and group II (SbRopGEF2, -6, -10), four members are in subfamily III (SbRopGEF4, -5, -7, -11), and one member is in group IV (SbRopGEF8). For ZmRopGEFs, three members are found in group I (ZmRopGEF1, -2, -13), four members in subfamily II (ZmRopGEF3, -4, -12, -15), six members in group III (ZmRopGEF5, -6, -8, -9, -11, -14), and two members in group IV (ZmRopGEF7, -10). These RopGEF proteins closely group with AtRopGEF proteins, suggesting that they are evolutionarily related to AtRopGEFs and may perform similar biological functions across species (Figure 2).

### 3.3. Conserved Motif Analysis and 3D Structure Prediction of the RopGEF Family Proteins

**Figure 3:**
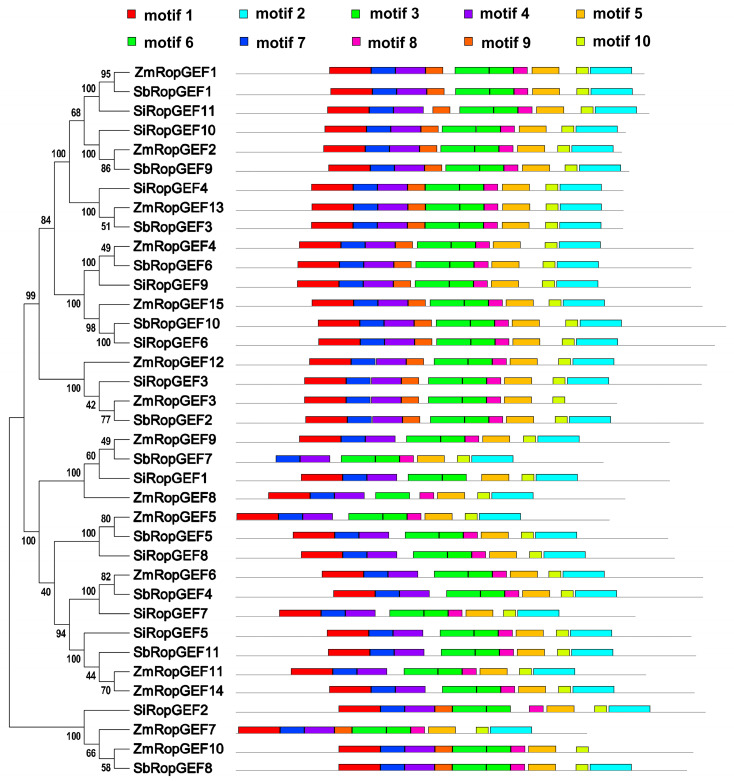
The motif distributions of RopGEF family proteins. Names of genes are indicated on the left. The motif distributions related to each of the RopGEFs are displayed on the right-hand side. The motifs, numbered 1–10, are displayed in different colored boxes. The sequence information for each motif is provided in Table S2.

To further investigate the architecture of the RopGEF family members, the conserved motifs of RopGEF proteins were sought through the online MEME server (Version 5.5.5, https://meme-suite.org/meme/tools/meme) with default parameters. A total of 10 distinct motifs were identified and numbered from motif 1 to motif 10. The details of the putative motifs are presented in Table S2. Based on the analysis results, motifs 3, 4, 5, 6, 7, 8, and 10 are widely distributed among all identified family members and constitute key motifs of the RopGEF family proteins. Meanwhile, similar motif composition and assembly order are conserved among members of the same subfamily of RopGEF (Figure 3). For instance, subfamilies I, II, and IV contained all of the ten motifs, arranged in the order of 1-7-4-9-6-3-8-5-10-2, except for ZmRopGEF3 and ZmRopGEF10 which lack motif 2. The order 1-7-4-6-3-8-5-10-2 appeared in subfamily III, except for SbRopGEF7, which lacked motif 1. It is worth noting that motif 9 does not occur in subfamily III (Figure 3 and Table S2). 

The 3D structure of these RopGEF proteins was further predicted using the Phyre2 Website. Results indicated that these 37 proteins in the three studied C_4_ Crops are structurally conserved and have extremely similar 3D structures (Figure S2). The 3D structures of these RopGEF proteins lay the foundation for their biological functions.

### 3.4. Exon–Intron Distribution of RopGEF Family Genes

**Figure 4:**
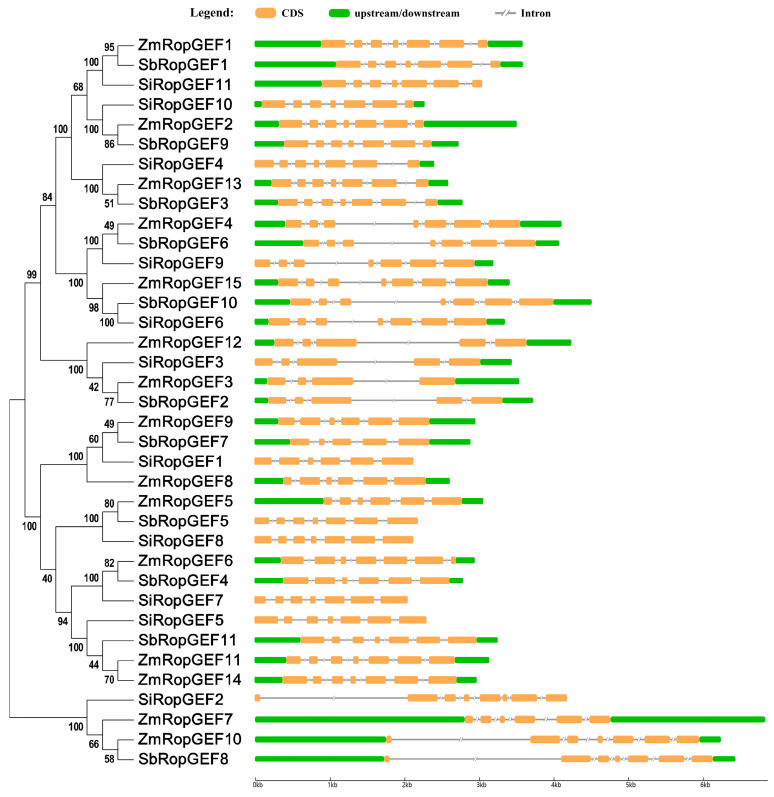
Exon–intron compositions of RopGEF family genes. Names of genes were indicated on the left. The exon–intron composition associated with each RopGEF is shown on the right. Exons are indicated by orange boxes, upstream/downstream is represented by green boxes, and introns are indicated by gray lines.

The *RopGEF* gene structure diagram was constructed based on the CDS sequence and genomic DNA sequence, which could clearly display the distribution position of each exon and intron in its own gene sequence. We discovered that intron–exon organization and the distribution of RopGEF family genes are similar, although there are individual differences. It was found that the number of introns in each of the studied genes ranged from three to seven (Figure 4). For example, all the genes in subfamily I contain six introns. The number of introns in genes varies significantly in subfamily II. A total of six of the ten genes contain six introns, three contain four introns, and one contains three introns. In subfamily III, the number of introns in genes also varies greatly. A total of eight of fourteen genes contain six introns, five genes contain five introns, and one gene contains four introns. Meanwhile, the gene structure is highly different in this subfamily. Five members (*SiRopGEF1*, *SiRopGEF5*, *SiRopGEF7*, *SiRopGEF8*, *SbRopGEF5*) did not have a 5′ UTR and 3′ UTR, while three members (*SiRopGEF3*, *SiRopGEF4*, *SiRopGEF9*) did not have a 5′ UTR, and one (*SiRopGEF11*) did not have a 3′ UTR. Among the four members in subfamily IV, *SiRopGEF2* has seven introns, *SbRopGEF8* and *ZmRopGEF10* have six introns, and *ZmRopGEF7* has five introns.

### 3.5. Tissue-Specific Expression Profiles of SiRopGEF Family Genes

**Figure 5:**
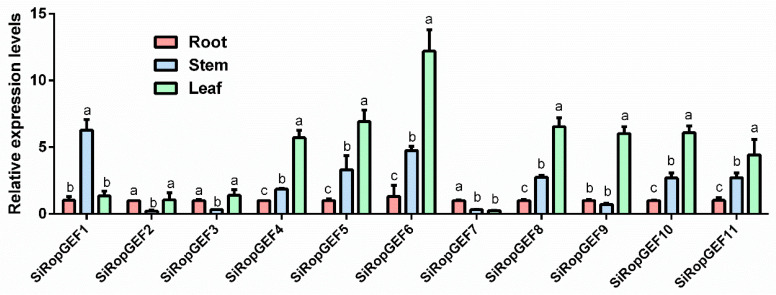
Tissue-specific expression pattern of *SiRopGEFs*. At the seedling stage (28 days), the millet plants were harvested to collect the roots, stems, and leaves for tissue-specific expression analysis of SiRopGEF family genes using qRT-PCR. The samples were immediately frozen in liquid nitrogen and stored at −80 °C for further analysis. The *x*-axis represents the different genes from various tissues while the *y*-axis displays the relative expression levels of each gene compared to the controls at the roots. The error bars indicate the standard deviations of the three independent biological replicates. Lowercase letters (a–c) denote significant differences (*p* < 0.05).

Transcriptional levels of 11 *SiRopGEFs* in three different tissues of millet seedlings were detected by qRT-PCR. The results showed that the expression levels of the majority of *SiRopGEFs* in leaf samples were significantly higher than those in roots, with the exception of *SiRopGEF7*. Furthermore, the transcript levels of the majority of *SiRopGEFs* in stems were intermediate between those in roots and leaves, with the exception of four genes (*SiRopGEF1*, *SiRopGEF2*, *SiRopGEF3,* and *SiRopGEF9*), where SiRopGEF1 had the highest transcript levels in stems and the other three had the lowest transcript levels in stems (Figure 5). These findings imply that the SiRopGEF family genes possess a distinct tissue-specific expression profile, indicating that there are both similarities and differences in the roles played by these *SiRopGEFs* in the growth of foxtail millet.

### 3.6. Transcriptional Profiles of SiRopGEF Family Genes under Abiotic Stresses and Phytohormone

**Figure 6:**
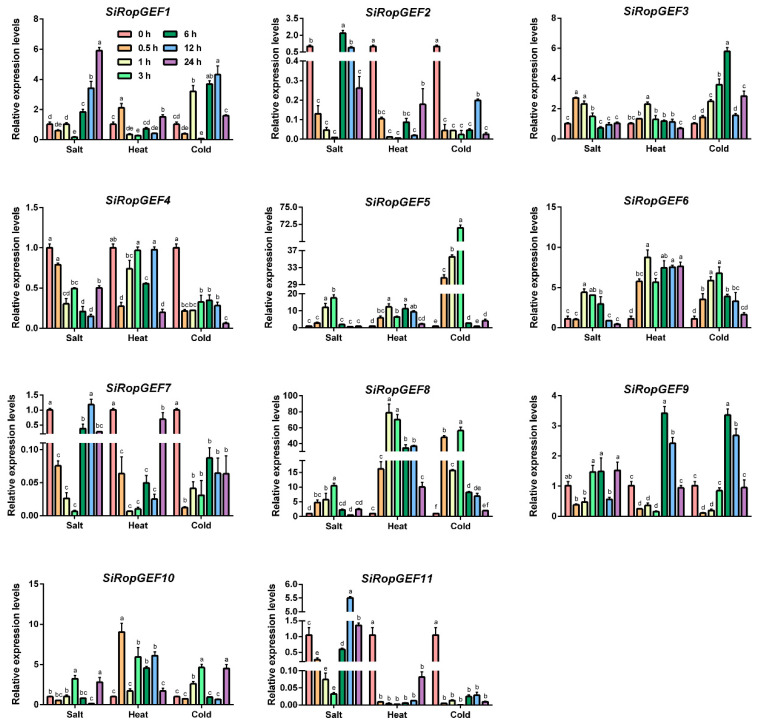
Transcriptional expression profiles of the *SiRopGEFs* when responding to abiotic stresses. Gene expression analysis was conducted using qRT-PCR to assess the impact of salt (200 mM NaCl), heat (40 °C), and cold (4 °C) stresses. Plants that were 4 weeks old were subjected to these stress conditions. The treatments are displayed on the *x*-axis, while the relative expression levels of the genes under each treatment compared to the controls (0 h) are shown on the *y*-axis. Error bars represent the standard deviations of three independent biological replicates. Significance levels (*p* < 0.05) are denoted by small letters (a–f).

To explore whether *SiRopGEFs* are implicated in the response to abiotic stresses, qRT-PCR analyses were conducted to investigate the transcript levels of all 11 *SiRopGEFs* under salt, heat and cold stresses. In Figure 6, it is evident that the transcription levels of *SiRopGEFs* undergo significant changes when exposed to salt, heat, and cold stresses. For instance, under salt stress, *SiRopGEF3*, *SiRopGEF5*, *SiRopGEF6,* and *SiRopGEF8* exhibited a gradual increase followed by a gradual decrease, with some fluctuations at specific time points. Conversely, *SiRopGEF1*, *SiRopGEF2*, *SiRopGEF4, SiRopGEF7,* and *SiRopGEF11* displayed a reverse trend of change. When subjected to heat stress, *SiRopGEF2*, *SiRopGEF7*, and *SiRopGEF11* experienced a sharp decrease after 0.5 h, then stabilized at lower levels before returning to pre-treatment levels after 24 h. *SiRopGEF3*, *SiRopGEF5*, and *SiRopGEF8*, on the other hand, showed an initial increase, peaked at 1 h, and then decreased. Under cold stress, *SiRopGEF2*, *SiRopGEF4*, *SiRopGEF7,* and *SiRopGEF11* sharply decreased after 0.5 h, then slowly increased. Meanwhile, *SiRopGEF3* and *SiRopGEF6* gradually increased, peaked at 3 or 6 h, and then slowly decreased. Despite the varying expression patterns, all *SiRopGEFs* experienced significant transcription level changes under salt, heat, and cold stresses, suggesting their involvement in plants’ response to abiotic stress.

**Figure 7:**
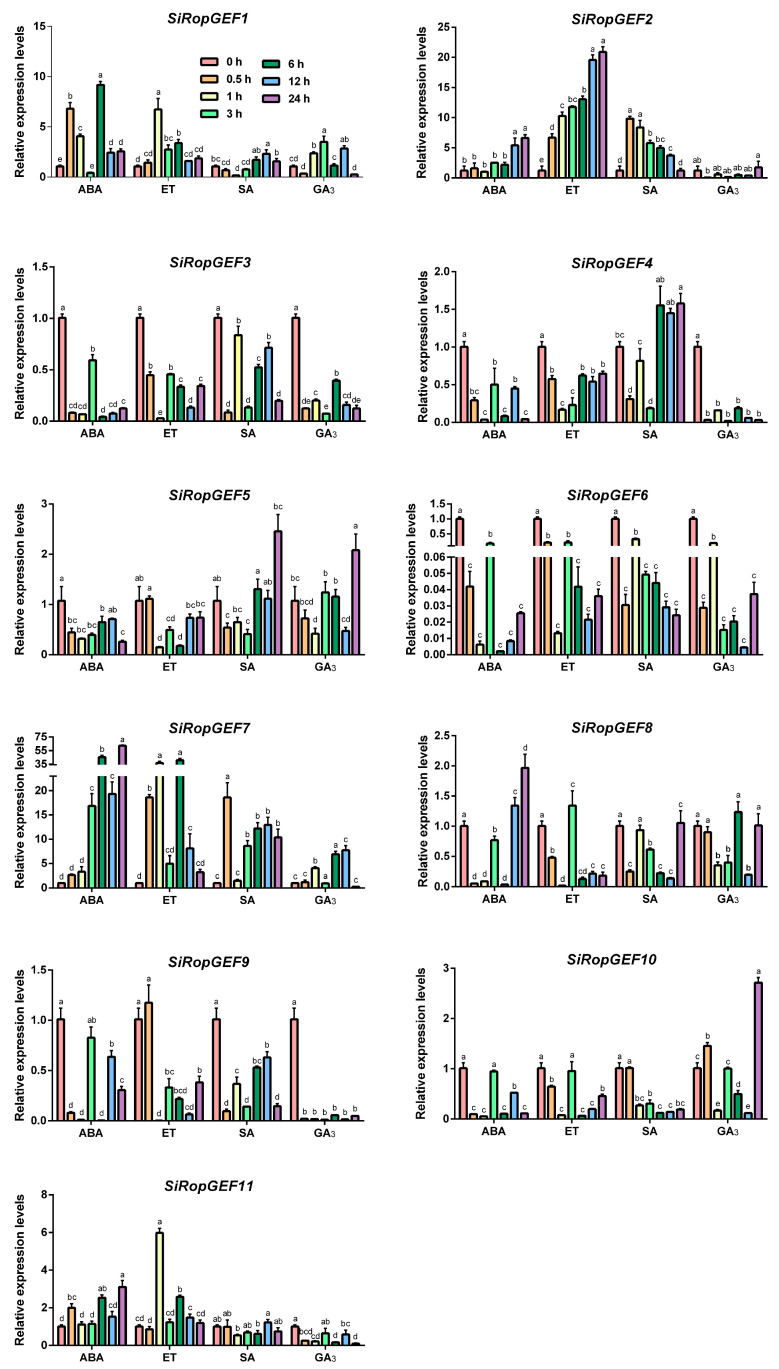
Transcriptional expression profiles of the *SiRopGEFs* in responding to hormonal applications. The gene expression profile was analyzed using qRT-PCR after treating 4-week-old plants with exogenous hormones such as ABA (100 μM), GA_3_ (100 μM), SA (500 μM), and ET (100 μM). The *x*-axis represents the different treatments, while the *y*-axis displays the relative expression levels of each gene compared to the controls (0 h). Error bars on the graph indicate the standard deviations of three independent biological replicates. Lowercase letters (a–e) denote statistically significant differences (*p* < 0.05).

Additionally, we conducted tests on the transcription levels of 11 *SiRopGEFs* after exogenous application of phytohormone ABA, ET, SA, and GA_3_ (Figure 7). *SiRopGEF2* and *SiRopGEF7* exhibited a continuous increase in transcription levels following the application of ABA, with *SiRopGEF7* showing a rapid increase at the 6 h time point. On the other hand, *SiRopGEF3*, *SiRopGEF4*, *SiRopGEF8*, *SiRopGEF9,* and *SiRopGEF10* experienced a sharp decrease in transcription levels after 0.5 h of ABA treatment, followed by fluctuations. The application of ET significantly upregulated the expression levels of *SiRopGEF2* at all the test time points. However, *SiRopGEF3*, *SiRopGEF4*, *SiRopGEF6*, *SiRopGEF8,* and *SiRopGEF10* saw a sharp decrease in expression levels after 1 h of exogenous ET application, followed by fluctuating transcription levels. When SA and GA_3_ were applied, the majority of SiRopGEFs showed decreased expression levels that slowly recovered. *SiRopGEF9*, in particular, maintained a low transcription level after GA_3_ treatment. These findings suggest that the members of the SiRopGEF family may play a role in hormone signaling pathways.

## 4. Discussion

Rop proteins function as a key component in signaling pathways, with their activity being modulated by RopGEFs, which facilitate the exchange of GDP for GTP to activate Rho GTPases [8]. These RopGEFs are a group of plant-specific proteins characterized by a conserved PRONE domain responsible for Rop binding and GEF function, along with variable *N*- and *C*-terminal regions [8]. The PRONE domain, located centrally, is crucial for catalyzing nucleotide exchange, while the N and C termini regulate GEF activity [8,29]. Research on RopGEFs in various plant species, such as *Arabidopsis*, *O. sativa*, *M. truncatula*, *B. rapa*, *S. lycopersicum*, and *P. patens* has highlighted their involvement in processes like plant growth, cell tip development, fertilization, and immune responses [8,12,13,14,15,16]. In this investigation, we have identified a total of 37 *RopGEFs*, comprising 11 *SiRopGEFs* from millet, 11 *SbRopGEFs* from sorghum, and 15 *ZmRopGEFs* from maize (Table 1 and Table S2). Our analysis focused on examining the structure, evolutionary relationships, and expression profiles of these *RopGEFs*. The primary objective was to uncover the functions of RopGEFs in plant growth, development, response to abiotic stress, and hormone signaling pathways.

### 4.1. The Genetic and Protein Architectures of RopGEF Family Members Demonstrate a Significant Relationship with Evolutionary Mechanisms, Suggesting the Possibility of Functional Redundancy among the Genes within the RopGEF Family

This study presents genome-wide identification of RopGEF family genes and their evolutionary relationships in three C_4_ Crops (millet, sorghum, and maize). The 37 RopGEFs are categorized into four subfamilies based on the phylogenetic tree, and the members of each subfamily share similar amino motif composition and exon–intron distribution. 

For example, all RopGEF members contain motifs 3, 4, 5, 6, 7, 8, and 10. The majority of subfamily I, II, and IV members possess all ten tested motifs, arranged in the order of 1-7-4-9-6-3-8-5-10-2. All subfamily III members lack motif 1, and the rest of the motifs 1-7-4-6-3-8-5-10-2, in order, appear in subfamily III, except for SbRopGEF7, which lacks motif 1 (Figure 3 and Table S2). These findings suggest that the protein structures of RopGEF family members have a strong correlation with evolutionary processes in C_4_ Crops. Additionally, the *RopGEF* genes exhibit a relatively uniform distribution of exons and introns within each subfamily (Figure 4). Subfamily I consist of genes with six introns each. Subfamilies II and III, on the other hand, have a range of 4–6 introns. The findings presented here offer evidence that the gene architectures of RopGEF family members are closely linked to the evolutionary mechanisms in C_4_ Crops.

The duplication of particular genes, chromosomal regions, or entire genomes is crucial to the evolutionary development of plant genomes. Mechanisms such as large-scale duplications and tandem duplications are vital for the proliferation of gene family members within the genome over the course of evolution. The gene and protein architectures of the RopGEF family reveal notable similarities, suggesting that gene duplication events may have occurred among members of the RopGEF family, which are closely associated with evolutionary dynamics. Generally, the sequence homology found between genes and proteins indicates a likelihood of similar higher-order structures, while the similarities in protein spatial arrangements imply the potential for analogous or overlapping biological functions. These findings suggest that functional redundancy may exist among RopGEF family members that possess highly similar sequences.

### 4.2. The Tissue-Specific Expression Patterns of SiRopGEF Family Genes Have Unveiled Various Roles in the Growth and Development of Foxtail Millet

In plants, the expression profiles of genes in specific tissues are intricately linked to their biological functions throughout the growth and development stages. Previous research has indicated that members of the RopGEF family play crucial roles in plant growth and root hair formation, as well as flower organ development. For instance, the necessity of AtRopGEF3 for the proper polarization of ROP and the efficient emergence of root hairs, as well as the involvement of AtRopGEF4 in the growth of root tips, has been demonstrated [30]. Additionally, OsRopGEF3 has been shown to interact with OsRac3 to regulate the elongation of root hairs and the formation of reactive oxygen species in rice [20]. Furthermore, the participation of OsRopGEF7B in the development of floral organs has been identified [23]. Moreover, the activation of OsRac1 by MtRopGEF10 to initiate the molecular signaling pathway for the formation of small cuticular papillae has been established [24]. And, the essential role of MtRopGEF2 in the development of root hairs has been confirmed [25].

In this study, we observed that the levels of expression of the majority of *SiRopGEFs* were notably elevated in the leaf in contrast to the roots and stems, while the transcription levels in the stems were found to be intermediate between those in the roots and leaves (Figure 5). Additionally, it was observed that the transcriptional patterns of *SiRopGEF4*, *SiRopGEF5*, *SiRopGEF6*, *SiRopGEF8*, *SiRopGEF10*, and *SiRopGEF11* were similar in millet tissue, while *SiRopGEF2* and *SiRopGEF3* exhibited comparable transcriptional patterns. These findings suggest that the *SiRopGEF* gene family likely plays crucial roles in the growth and development of millet, with certain genes potentially sharing similar biological functions in the growth and development of millet.

### 4.3. The Inducible Expression Pattern of SiRopGEF Family Genes Suggests a Crucial Role in Abiotic Stress and Hormone Signaling

Different environmental stressors like salinity, drought, and extreme temperatures can have detrimental effects on plant growth and development, leading to specific changes in the transcriptional levels of resistance-related genes. Our study revealed that the transcription levels of SiRopGEF family members were significantly upregulated under abiotic stresses such as salt, cold, and heat (Figure 6). For instance, the transcription levels of *SiRopGEF3*, *SiRopGEF5*, *SiRopGEF6* and *SiRopGEF8* were induced by salt stress, peaking briefly before declining, whereas *SiRopGEF1*, *SiRopGEF2*, *SiRopGEF4*, *SiRopGEF7* and *SiRopGEF11* exhibited an opposite pattern. After 0.5 h of heat stress, the transcription levels of *SiRopGEF2*, *SiRopGEF7*, and *SiRopGEF11* dropped sharply before stabilizing at a lower level, eventually returning to pre-stress levels after 24 h. Additionally, the transcription levels of *SiRopGEF2*, *SiRopGEF4*, *SiRopGEF7* and *SiRopGEF11* decreased initially before gradually increasing, while *SiRopGEF3*, *SiRopGEF6* and *SiRopGEF8* showed a contrasting trend. These findings suggest that members of the SiRopGEF family may play a role in plant responses to abiotic stress.

Plant hormones play a crucial role in regulating various processes in plant life, including growth, development, and defense mechanisms. Studies have shown that members of the SiRopGEF family are associated with multiple hormonal pathways. For instance, in *Arabidopsis*, RopGEF2 is believed to have a negative impact on seed germination and post-germination growth suppressed by ABA [31]. Additionally, ROPGEF1 and ROPGEF4 act as functional regulators of ROP11 GTPase in ABA-induced stomatal closure in *Arabidopsis* [15]. Moreover, RopGEF7 is thought to integrate positional information derived from auxin in a feed-forward mechanism, thereby controlling the maintenance of root stem cell niches by regulating PLETHORA transcription factors [16]. Our research has revealed that the transcription levels of SiRopGEF family members are significantly influenced by exogenous treatments of ABA, ET, SA, and GA_3_ (Figure 7). Following ABA treatment, the expression levels of *SiRopGEF3*, *SiRopGEF4*, *SiRopGEF8*, *SiRopGEF9,* and *SiRopGEF10* decreased sharply within 0.5 h, subsequently fluctuating. Similarly, exogenous ET application led to a rapid decrease in the expression levels *SiRopGEF3*, *SiRopGEF4*, *SiRopGEF6*, *SiRopGEF8,* and *SiRopGEF10* after 1 h, followed by fluctuating levels. Treatment with exogenous SA and GA_3_ resulted in decreased expression levels of most *SiRopGEFs*, which then slowly recovered. These findings suggest that members of the SiRopGEF family are likely involved in hormone signaling pathways.

## 5. Conclusions

A total of 37 *RopGEF* members were identified in the genomes of millet, sorghum, and maize. Subsequently, they were reclassified based on their chromosomal distribution and grouped into four subfamilies according to their phylogenetic relationships. Upon analyzing the structural characteristics of the *RopGEF* family members, it was noted that each subfamily exhibits similar structural features, including motif composition and conserved intron–exon distribution. The variations in the expression patterns of RopGEF members suggest their potential roles in plant growth and development, response to abiotic stress, and hormone signal transduction. These findings indicate that the *RopGEF* gene could serve as a valuable genetic marker for facilitating future research aimed at elucidating the functional traits of *RopGEFs*.

## Figures and Tables

**Table 1:** Identification of RopGEF family members in millet, sorghum, and maize.

Name	Gene ID	Genomic Location	Orientation	DNA	mRNA	PROTEIN	Exons
*SiRopGEF1*	SETIT_019913mg	Chr I: 10,428,890–10,430,997	Forward	2108	1560	519	6
*SiRopGEF2*	SETIT_020060mg	Chr I: 35,729,463–35,733,658	Reverse	4196	1689	562	8
*SiRopGEF3*	SETIT_029392mg	Chr II: 38,985,249–38,988,693	Reverse	3445	2097	557	5
*SiRopGEF4*	SETIT_029765mg	Chr II: 40,550,390–40,552,845	Reverse	2456	1591	463	7
*SiRopGEF5*	SETIT_021676mg	Chr III: 10,725,845–10,728,180	Forward	2336	1638	545	7
*SiRopGEF6*	SETIT_021607mg	Chr III: 17,936,647–17,939,988	Reverse	3342	2154	573	7
*SiRopGEF7*	SETIT_001271mg	Chr V: 32,966,428–32,968,465	Forward	2038	1437	478	7
*SiRopGEF8*	SETIT_001004mg	Chr V: 37,519,096–37,521,213	Forward	2118	1578	525	7
*SiRopGEF9*	SETIT_000923mg	Chr V: 41,473,739–41,476,975	Reverse	3237	1891	544	7
*SiRopGEF10*	SETIT_010033mg	Chr VII: 26,959,231–26,961,508	Forward	2278	1647	466	7
*SiRopGEF11*	SETIT_035358mg	Chr IX: 39,178,504–39,181,535	Reverse	3032	2380	494	7
*SbRopGEF1*	SORBI_3001G305300	Chr 1: 58,918,023–58,921,635	Forward	3613	2870	489	7
*SbRopGEF2*	SORBI_3002G284100	Chr 2: 66,489,622–66,493,393	Reverse	3772	2281	559	5
*SbRopGEF3*	SORBI_3002G302800	Chr 2: 67,888,132–67,890,903	Reverse	2772	2037	463	7
*SbRopGEF4*	SORBI_3003G254200	Chr 3: 59,270,205–59,273,022	Forward	2818	2251	559	6
*SbRopGEF5*	SORBI_3003G303200	Chr 3: 63,374,869–63,377,084	Forward	2216	1554	517	7
*SbRopGEF6*	SORBI_3003G356600	Chr 3: 67,492,758–67,496,854	Reverse	4097	2609	545	7
*SbRopGEF7*	SORBI_3004G123100	Chr 4: 13,838,770–13,841,653	Reverse	2884	2346	440	5
*SbRopGEF8*	SORBI_3004G269100	Chr 4: 61,343,743–61,350,249	Forward	6473	3669	540	7
*SbRopGEF9*	SORBI_3006G176400	Chr 6: 53,167,615–53,170,360	Forward	2746	2179	471	7
*SbRopGEF10*	SORBI_3009G159600	Chr 9: 51,718,034–51,722,569	Forward	4536	2753	586	7
*SbRopGEF11*	SORBI_3009G229900	Chr 9: 57,042,535–57,045,813	Reverse	3279	2558	550	7
*ZmRopGEF1*	Zm00001eb022950	Chr 1: 89,146,259–89,149,885	Reverse	3627	2678	489	9
*ZmRopGEF2*	Zm00001eb074280	Chr 2: 19,660,011–19,663,478	Reverse	3468	2940	462	7
*ZmRopGEF3*	Zm00001eb104060	Chr 2: 203,405,080–203,408,547	Reverse	3468	2374	456	4
*ZmRopGEF4*	Zm00001eb147940	Chr 3: 184,479,653–184,483,747	Forward	4095	2607	548	7
*ZmRopGEF5*	Zm00001eb152460	Chr 3: 200,467,395–200,470,469	Reverse	3075	2394	447	8
*ZmRopGEF6*	Zm00001eb157000	Chr 3: 215,085,961–215,088,881	Reverse	2921	2279	559	7
*ZmRopGEF7*	Zm00001eb188240	Chr 4: 164,693,645–164,700,513	Reverse	6869	3164	420	10
*ZmRopGEF8*	Zm00001eb203460	Chr 4: 228,169,534–228,172,117	Reverse	2584	2102	466	6
*ZmRopGEF9*	Zm00001eb237470	Chr 5: 138,168,337–138,171,296	Reverse	2960	2495	519	6
*ZmRopGEF10*	Zm00001eb251920	Chr 5: 208,547,083–208,553,330	Reverse	6248	2288	547	9
*ZmRopGEF11*	Zm00001eb295640	Chr 6: 175,617,292–175,620,402	Reverse	3111	2352	491	7
*ZmRopGEF12*	Zm00001eb321170	Chr 7: 153,963,165–153,967,384	Forward	4220	2554	564	5
*ZmRopGEF13*	Zm00001eb321410	Chr 7: 155,027,802–155,030,401	Reverse	2600	1890	464	7
*ZmRopGEF14*	Zm00001eb343580	Chr 8: 69,946,147–69,949,185	Reverse	3039	2292	549	7
*ZmRopGEF15*	Zm00001eb347730	Chr 8: 97,793,292–97,796,708	Reverse	3417	2286	558	7

## Data Availability

The original contributions presented in the study are included in the article/Supplementary Materials, further inquiries can be directed to the corresponding author.

## Supplementary Materials

The following supporting information can be downloaded at: https://www.mdpi.com/article/10.3390/genes15091112/s1, Table S1. Analysis of physicochemical properties of RopGEF family proteins in C4 Crops. Table S2. The conserved motif analysis of the RopGEF family proteins. Table S3. Primer sequences for qRT-PCR used in the study. Figure S1. The exploration of protein domains of RopGEF family proteins. Figure S2. The 3D structure modeling of RopGEF family proteins.
