# Supplementary material for: Genome-Wide Identification and Characterization of RopGEF Gene Family in C4 Crops
Source: Genes (Basel). 2024 Aug 23;15(9):1112. doi: 10.3390/genes15091112 (PMC11431098; doi:10.3390/genes15091112)
Supplement: Supplementary file 1 [file genes-15-01112-s001.zip › Table S1.pdf]

Table S1. Analysis of physicochemical properties of RopGEF family proteins in C_4 Crops.

Name	MW (Da)	pI	Charge	II	AI	GRAVY	Amino Acid Comosition %
SiRopGEF1	57981.64	5.94	-2	50.13	75.65	-0.55	Leu (10.0), Ala (9.6), Glu (8.5), Ser (8.3)
SiRopGEF2	62707.13	5.87	-2.5	49.12	90.05	-0.197	Leu (10.7), Ser (9.4), Lys (7.3), Ala (6.9)
SiRopGEF3	60678.89	5.74	-3	54.91	79.93	-0.314	Ser (12.9), Ala (9.5), Leu (9.5), Glu (7.2)
SiRopGEF4	52425.37	6	-1	60.59	73.74	-0.541	Ser (12.1), Leu (9.7), Glu (7.3), Ala (6.0), Asp (6.0)
SiRopGEF5	60294.45	6.63	3.5	55.64	78.4	-0.489	Ser (11.0), Leu (9.7), Ala (7.9), Asp (7.3)
SiRopGEF6	62459.8	5.81	-3	47.36	80.96	-0.338	Ala (11.0), Ser (9.8), Leu (9.4), Asp (7.2), Gly (7.2)
SiRopGEF7	53421.17	5.36	-11	50.65	88.39	-0.486	Leu (10.5), Asp (8.8), Ser (8.8), Ala (8.4)
SiRopGEF8	58549.13	5.22	-11	55.09	77.9	-0.613	Leu (9.7), Ala (8.8), Glu (8.4), Ser (8.2)
SiRopGEF9	60323.59	5.92	-2	59.54	77.72	-0.404	Ser (10.5), Leu (9.6), Ala (9.4), Glu (7.5)
SiRopGEF10	51592.48	6.01	-1	57.31	76.05	-0.481	Leu (9.7), Ser (9.7), Ala (9.0), Asp (7.5)
SiRopGEF11	53794.94	5.33	-7.5	48.15	80	-0.235	Ser (13.4), Ala (9.5), Leu (9.3), Asp (8.5)
SbRopGEF1	53078	5.79	-2.5	48.16	82	-0.243	Ser (13.1), Ala (9.6), Leu (9.2), Asp (8.4)
SbRopGEF2	60757.77	5.81	-2.5	53.64	77.35	-0.349	Ser (13.4), Leu (9.3), Ala (8.8), Glu (6.8)
SbRopGEF3	52332.12	6	-1	59.28	74.99	-0.526	Ser (13.0), Leu (9.3), Glu (7.3), Ala (5.8), Arg (5.8)
SbRopGEF4	61684.17	5.94	-2	66.84	77.17	-0.509	Ser (12.3), Ala (9.1), Leu (8.9), Arg (7.7)
SbRopGEF5	57435.99	5.42	-7	54.85	76.05	-0.626	Leu (9.3), Asp (8.5), Ser (8.1), Ala (7.7)
SbRopGEF6	60258.42	6.23	1	57	75.82	-0.416	Ser (10.1), Ala (9.9), Leu (9.2), Arg (7.2)
SbRopGEF7	49703.81	5.4	-7	50.35	79.89	-0.439	Leu (10.0), Ala (9.8), Glu (8.9), Lys (8.0)
SbRopGEF8	60252.24	6.13	0	48.83	88.48	-0.25	Leu (10.2), Ser (9.8), Asp (7.2), Lys (7.2)
SbRopGEF9	52042.95	5.89	-2	62.56	75.22	-0.476	Ser (9.8), Leu (9.6), Ala (8.5), Arg (7.4), Glu (7.4)
SbRopGEF10	63440.64	5.71	-4	46.96	80.97	-0.303	Ala (11.1), Ser (11.1), Leu (8.4), Gly (7.2)
SbRopGEF11	60919.22	7.13	4.5	58.56	77.69	-0.513	Ser (11.1), Leu (9.6), Ala (7.6), Lys (6.9)
ZmRopGEF1	53195.1	6.06	-0.5	47.89	80.82	-0.276	Ser (13.9), Ala (9.4), Leu (9.4), Asp (8.8)
ZmRopGEF2	51466.64	5.94	-1.5	61.03	80.06	-0.417	Leu (10.0), Ser (9.3), Ala (9.1), Asp (7.6)4
ZmRopGEF3	50080.22	5.67	-3	48.84	80.48	-0.249	Ser (12.5), Leu (10.5), Ala (8.3), Glu (6.8)

ZmRopGEF4	60645.18	6.27	1	57.33	78.21	-0.374	Ser (9.9), Leu (9.5), Ala (9.1), Arg (6.9), Glu (6.9)
ZmRopGEF5	50177.95	5.37	-7	47.85	81.86	-0.575	Leu (10.5), Asp (8.7), Ala (7.6), Lys (7.6)
ZmRopGEF6	61647.22	5.67	-6	63.18	79.96	-0.529	Ser (10.6), Leu (9.3), Ala (8.9), Asp (8.8)
ZmRopGEF7	47376.1	9.03	10	51.61	92.86	-0.198	Leu (10.7), Ser (8.1), Lys (7.6), Ala (7.4), Val (7.4)
ZmRopGEF8	51877.93	5.59	-4.5	51.92	76.72	-0.491	Leu (10.5), Ala (10.1), Ser (9.0), Glu (8.2)
ZmRopGEF9	58204.05	5.93	-2	50.59	78.81	-0.515	Leu (10.8), Ala (9.1), Ser (8.7), Glu (7.9)
ZmRopGEF10	61130.08	5.91	-2	48.46	86.97	-0.288	Le (u (9.9), Ser (9.7), Asp (7.1), Lys (6.9)
ZmRopGEF11	53826.02	6.2	0.5	49.72	82.28	-0.45	Ser (10.6), Leu (10.0), Ala (9.4), Asp (7.3)
ZmRopGEF12	61436.64	6.25	0	55.49	77.2	-0.351	Ser (13.8), Leu (9.4), Ala (8.9), Glu (6.9)
ZmRopGEF13	52409.56	5.95	-1.5	60.89	76.9	-0.441	Ser (13.4), Leu (9.5), Glu (7.3), Lys (6.0)
ZmRopGEF14	60625.12	8.12	7	55.07	80.18	-0.431	Ser (10.0), Ala (9.7), Leu (9.7), Arg (7.1)
ZmRopGEF15	60465.65	6.36	1.5	49.97	84.01	-0.241	Ala (12.0), Ser (10.6), Leu (9.0), Asp (7.3)

List of abbreviation: MW, Molecular weight; pI, Theoretical isoelectric point; II, Instability index; AI, Aliphatic index; GRAVY, Grand average of hydropathicity.
