# Supplementary material for: Genome-Wide Identification and Characterization of RopGEF Gene Family in C4 Crops
Source: Genes (Basel). 2024 Aug 23;15(9):1112. doi: 10.3390/genes15091112 (PMC11431098; doi:10.3390/genes15091112)
Supplement: Supplementary file 1 [file genes-15-01112-s001.zip › Table S2.pdf]

Table S2. The conserved motif analysis of the RopGEF family proteins.

Motifs No.	sequences	E-value	Sites	Width	Logo
1	MKEKFAKLLLGEDMSGSGKGVCTALAJSNAITNLAATVFGELRRLEPLAP	9.5e-1441	37	50	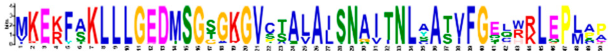
2	KHRFPGLPQTTLDISKIQYNKDVGQAILESYSRVLESLAFNIVSRIDDVL	9.7e-1296	35	50	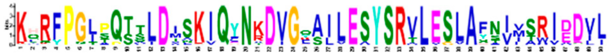
3	KWWLPVPRVPPEGLSDATRKWLQHQRDCANQILKAAMAINSN	1.3e-1049	37	42	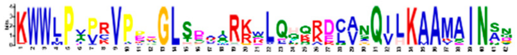
4	EKKAMWRREMDWLLSVADHIVEFVPTKQTLPBGT	8.8e-822	37	34	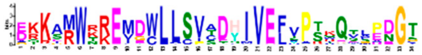
5	DQFSPEELLDCLDLSSEHKALEJABRVEASVYVW	6.1e-711	37	34	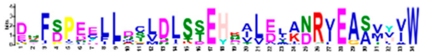
6	EVMTTRPRSDLYMNJPALRKLDAMLLEIL	1.4e-699	37	29	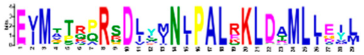
7	EMEIPESYLESLPKNGRASLGDSIYRYIT	1.2e-584	34	29	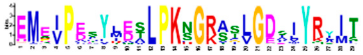
8	EFWYVDQGSRAPDSD	6.7e-223	37	15	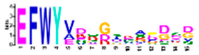
9	EKREMLAERAETLLL	4.5e-191	37	15	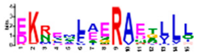
10	STKDSKSSWGSVKDL	8.4e-103	33	15	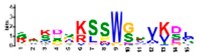
