# Supplementary material for: Genome-Wide Identification and Characterization of RopGEF Gene Family in C4 Crops
Source: Genes (Basel). 2024 Aug 23;15(9):1112. doi: 10.3390/genes15091112 (PMC11431098; doi:10.3390/genes15091112)
Supplement: Supplementary file 1 [file genes-15-01112-s001.zip › Table S3.pdf]

Table S3. Primer sequences for qRT-PCR used in the study.

Gene Name	Primer Name	Primer Sequences
SETIT_019913mg	*qSiRopGEF1*-F	TGCTCATCGGCTACATGGAC
	qSiRopGEF1-R	AAGGTTTGCCTTTGGGAGCG
SETIT_020060mg	*qSiRopGEF2*-F	GCAAGCTTTGAGCTTCACGTT
	qSiRopGEF2-R	TTCCAAGCAAACACTGCACC
SETIT_029392mg	*qSiRopGEF3*-F	AGAGCACCACACACTCGAAG
	qSiRopGEF3-R	CCCTTGACTTTTCCACCCCA
SETIT_029765mg	*qSiRopGEF4*-F	CATGAGGCGCTTGAGATTGC
	qSiRopGEF4-R	GCTCGGTAACGTTCTCCCAT
SETIT_021676mg	*qSiRopGEF5*-F	AACGGGACCTGCATGGAAAT
	qSiRopGEF5-R	CCAGAACTCCTGCTTGTCGT
SETIT_021607mg	*qSiRopGEF6*-F	AGTACAACAAGGACGTGGGG
	qSiRopGEF6-R	GATTGCTTGCTCAGCTCGTC
SETIT_001271mg	*qSiRopGEF7*-F	GCCAAAGAATGGGAGGTCCA
	qSiRopGEF7-R	CTCGCTTCTCCAAGCTGACA
SETIT_001004mg	*qSiRopGEF8*-F	GGTCTGACGAGAAGTGGTGG
	qSiRopGEF8-R	ATGAGGCAGTTGGCGTTGAT
SETIT_000923mg	*qSiRopGEF9*-F	GAGTGCCTTCTTGACTGCCT
	qSiRopGEF9-R	ATCTAGCACTGCTGCTACGG
SETIT_010033mg	*qSiRopGEF10*-F	CGAGAAATGGACTGCCTGCT
	qSiRopGEF10-R	TTGACGTAGATGTCGGGCCT
SETIT_035358mg	*qSiRopGEF11*-F	CTCACGAGTCACAATCGCCT
	qSiRopGEF11-R	ATCCATCGAAACGGGAGACG
