# Supplementary figures and images for: Genome-Wide Identification and Characterization of RopGEF Gene Family in C4 Crops
Source: Genes (Basel). 2024 Aug 23;15(9):1112. doi: 10.3390/genes15091112 (PMC11431098; doi:10.3390/genes15091112)

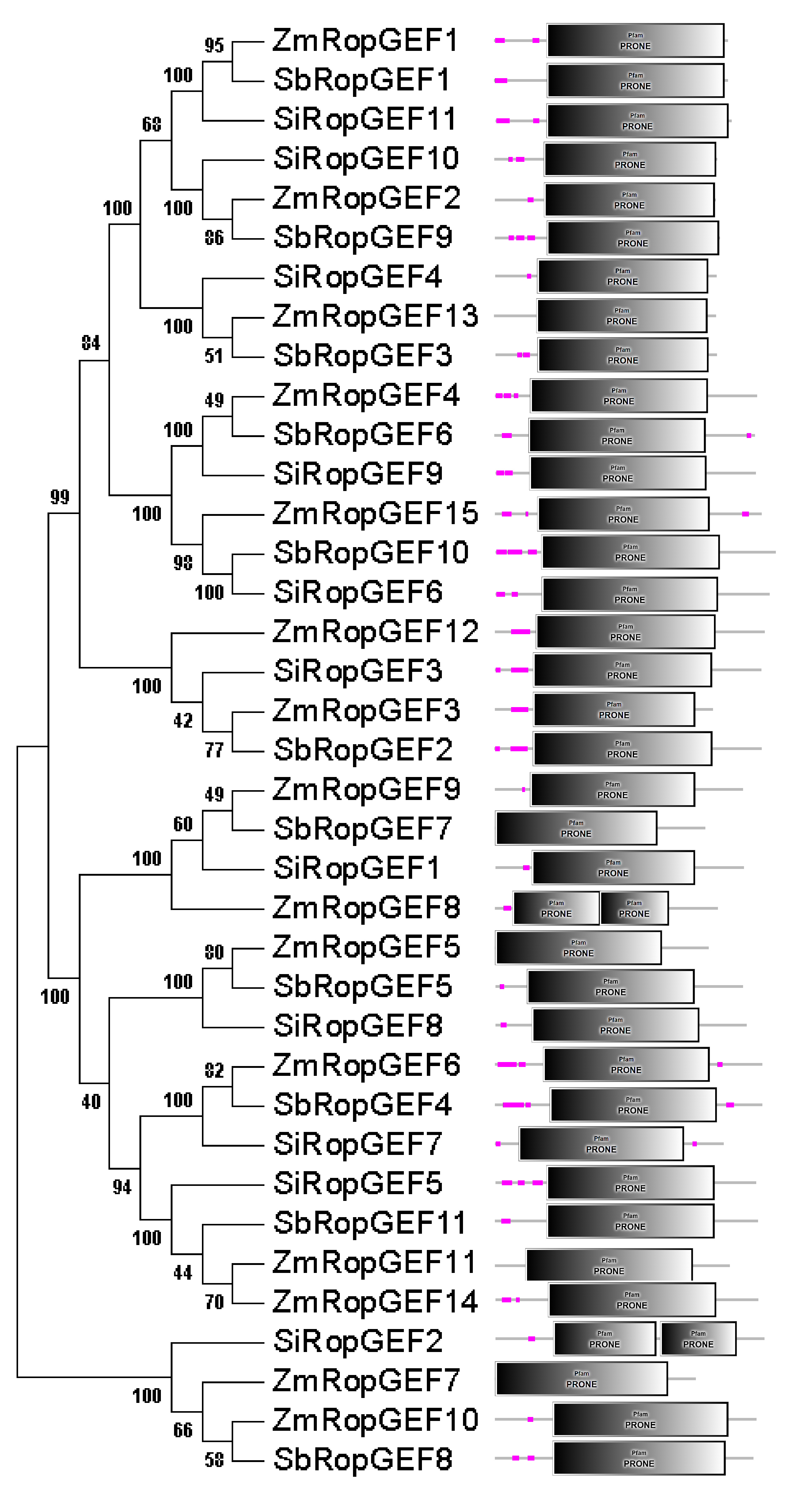

Supplement: Supplementary file 1 [file genes-15-01112-s001.zip › Figure S1.tif]

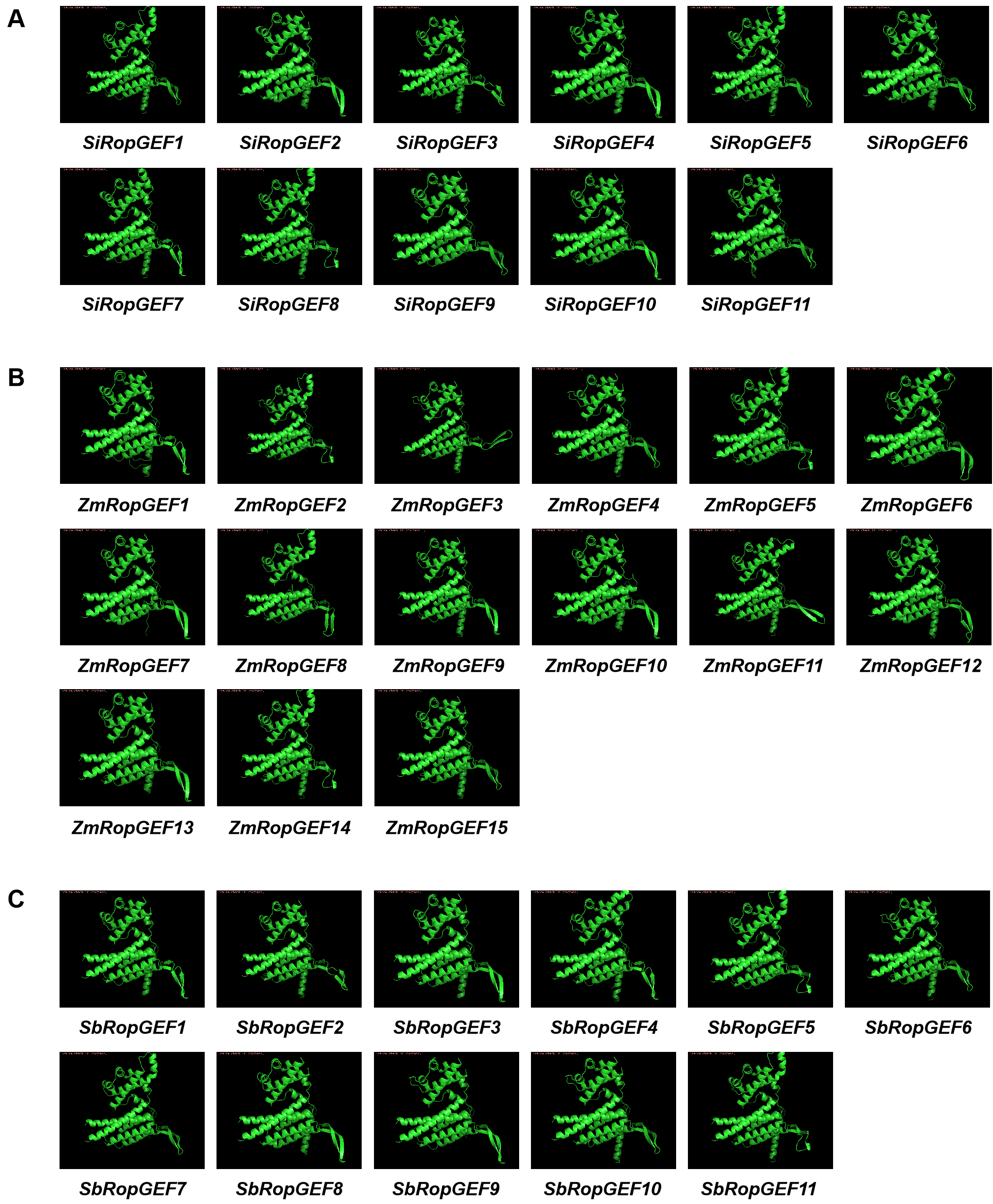

Supplement: Supplementary file 1 [file genes-15-01112-s001.zip › Figure S2.png]
